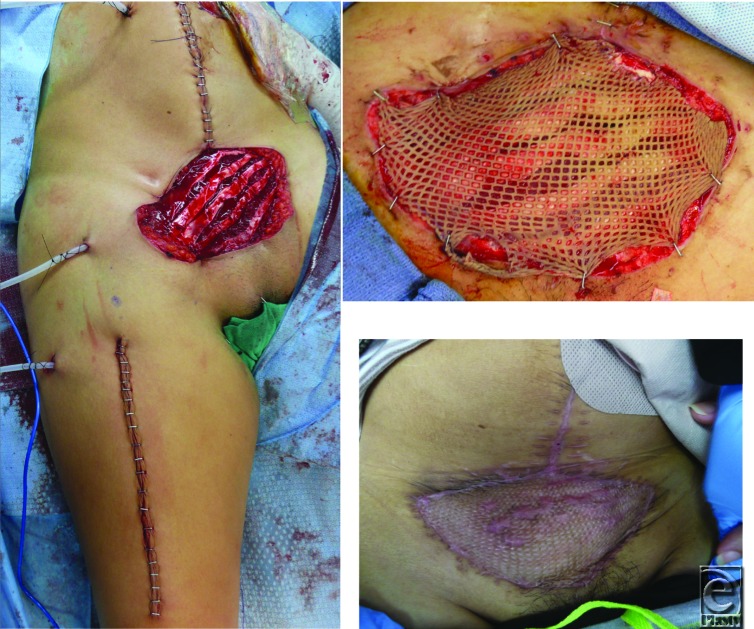# Abdominal Wall Reconstruction

**Published:** 2013-01-21

**Authors:** Michael Ingargiola, Lily Daniali, Edward Lee, Mark Granick

**Affiliations:** Division of Plastic Surgery, Department of Surgery, New Jersey Medical School—University of Medicine and Dentistry of New Jersey, Newark, NJ

**Figure:**
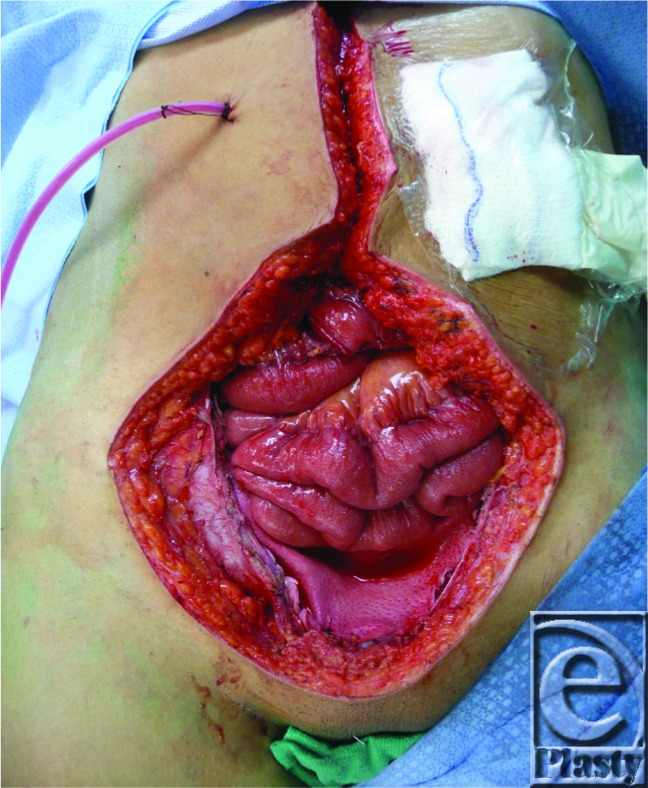


## DESCRIPTION

A 39-year-old woman presented with abdominal pain, hematuria, and painful infraumbilical mass. She had infiltration of her bilateral rectus muscles, uterus, bladder, and rectum by a moderately differentiated adenocarcinoma of urachal origin. After total pelvic exenteration, creation of an ileal conduit, and ileocolostomy for palliative tumor debulking, an abdominal wall defect extending from bilateral anterior iliac spines and a height of 10 cm from supraumbilical to the symphysis pubis remained.

## QUESTIONS

1. **What is an accepted algorithm for analyzing abdominal wall defects requiring reconstruction?**
2. **What are the benefits and concerns with using prosthetic or biologic material in this situation?**
3. **What reconstructive options are available for immediate reconstruction of the abdominal wall?**

## DISCUSSION

Abdominal wall reconstruction requires anatomic defect analysis, evaluation of reconstructive goals, and use of many plastic surgical techniques and reconstructive strategies to restore abdominal wall integrity and cosmesis. Assessment of the wound bed for inflammation, infection, neoplasm, trauma, and previous surgeries is of primary importance to determining the timing and technique of reconstruction.^1^ Inflammation may limit the ability to advance tissues, and the combination of inflammation and contamination may require delay of reconstruction. In contaminated wounds, temporizing or staged reconstructions may be necessary. Contaminated wound beds are poor candidates for prosthetic mesh placement secondary to high rates of mesh infection, fistula formation, and extrusion.^2^^,^^3^ In such a setting, biologic material, such as acellular, human, or porcine dermal matrices may be used as an alternative.^5^^,^^6^ When placed between the abdominal wall and underlying bowel, animal studies have demonstrated a decrease incidence of abdominal adhesions.^7^ While lacking the tensile strength of prosthetic mesh, they are more resistant to infectious complications due to an ability to revascularize and become infiltrated by host fibroblast cells.^8^^-^10

Abdominal wall defect analysis has 3 essential components: defect size, location on the abdominal wall (upper, middle, and/or lower third; central or lateral), and tissue requirements for reconstruction (partial vs full-thickness defect).^1^ Partial defects of skin and subcutaneous tissue only can be reconstructed with primary closure if less than 5 cm at maximum width. Skin grafting, local flaps, vacuum-assisted closure (VAC) devices, or tissue expansion may be used depending on the size of the defect.

The method of repair of partial defects of the abdominal wall muscle and fascia is dictated primarily by the position of the defect and the importance of establishing a tension-free closure to prevent future fascial dehiscence. Midline myofascial defects may be amenable to reapproximation with component separation using rectus abdominis muscle. Bilateral relaxing fascial releases provide a total of 10, 18, and 6 to 10 cm of advancement in the upper, middle, and lower thirds of the abdomen, respectively.^11^ Large complete defects of the midline often require local or distant flaps for reconstruction of the abdominal wall, coupled with either skin grafting or tissue expansion for skin coverage. Flap choice is dictated by the position of the defect on the abdominal wall and ability of the flap to reach without tension.

Reconstruction of midline defects with local flaps and distant lateral flaps is often limited by poor advancement and arc of rotation of the abdominal wall. The superiorly or inferiorly based rectus abdominis muscle flap emerges as the workhorse for the reconstruction of abdominal wall defects. Its arc of rotation is excellent for lateral defects, but its use for midline defects as a turnover flap has also been well described.^12^ Bilateral external oblique flaps may be advanced for reconstruction of the upper two thirds of the abdominal wall. Its arc of rotation and unreliable blood supply limit its use for the lower third of the abdominal wall.^13^ The tensor fasciae latae and rectus femoris flaps are 2 of the major lower extremity muscle flaps available for lower midline abdominal wall reconstruction. The tensor fasciae latae muscle is small and does not provide adequate bulk for large volume defects. Furthermore, the distal cutaneous extension of the flap is limited due to the risk of lateral knee instability.

In this case, a rectus femoris flap was selected primarily for its reliable vascularity, arc of rotation, and ability to provide adequate width and muscle bulk for a large volume defect. The reconstruction of the lower abdominal wall defect was reinforced with an underlay of human acellular dermal matrix. In a second stage, a split-thickness skin graft was placed. The patient recovered well postoperatively without wound or donor site complications.

## Figures and Tables

**Figure:**